# A Prediction Nomogram for No-Reflow in Acute Myocardial Infarction Patients after Primary Percutaneous Coronary Intervention

**DOI:** 10.31083/j.rcm2505151

**Published:** 2024-04-30

**Authors:** Bowen Lou, Kejia Kan, Hui Liu, Rilu Feng, Xinyu Zhang, Zuyi Yuan, Lan Zhang, Jianqing She

**Affiliations:** ^1^Cardiovascular Department, First Affiliated Hospital of Xi’an Jiaotong University, 710061 Xi’an, Shaanxi, China; ^2^Key Laboratory of Environment and Genes Related to Diseases, Ministry of Education, 710061 Xi’an, Shaanxi, China; ^3^Department of Vascular Surgery, Renji Hospital, Shanghai Jiao Tong University School of Medicine, 200127 Shanghai, China; ^4^Biobank, First Affiliated Hospital of Xi’an Jiaotong University, 710061 Xi’an, Shaanxi, China; ^5^Department of Endocrinology and Metabolism, Renji Hospital, School of Medicine, Shanghai Jiao Tong University, 200127 Shanghai, China

**Keywords:** no-reflow (NR), acute myocardial infarction (AMI), primary percutaneous coronary intervention (pPCI), prediction nomogram

## Abstract

**Background:**

The coronary no-reflow (NR) phenomenon is an independent predictor of major adverse cardiac events (MACEs). This study aimed to establish a clinical and comprehensive nomogram for predicting NR in acute myocardial infarction (AMI) patients after primary percutaneous coronary intervention (pPCI).

**Methods:**

The multivariable logistic regression analysis was performed to determine the NR-related factors. A nomogram was established via several clinical and biochemical factors, and the performance was evaluated via discrimination, calibration, and clinical factors.

**Results:**

The study consisted of 3041 AMI patients after pPCI, including 2129 patients in the training set (70%) and 912 patients in the validation set (30%). The NR event was 238 in the training set and 87 in the validation set. The level of N-terminal prohormone B-type natriuretic peptide (NT-proBNP), basophil count (BASO), neutrophil count (NEUBC), D-dimer, hemoglobin (Hb), and red blood cell distribution width (RDW.CV) in NR patients showed statistically significant differences. In the training set, the C-index was 0.712, 95% CI 0.677 to 0.748. In the validation set, the C-index was 0.663, 95% CI 0.604 to 0.722.

**Conclusions:**

A nomogram that may predict NR in AMI patients undergoing pPCI was established and validated. We hope this nomogram can be used for NR risk assessment and clinical decision-making and significantly prevent potentially impaired reperfusion associated with NR.

## 1. Introduction

Assessing coronary flow is the top priority after it has been verified that there is no residual stenosis following primary percutaneous coronary intervention (pPCI) for an acute myocardial infarction (AMI). Many of the well-known risk factors associated with the no-reflow (NR) phenomenon are common risk factors for cardiovascular diseases, such as smoking, hypertension, dyslipidemia, diabetes, and hemodynamic instability [1, 2, 3]. However, there is no general consensus on the correct prevention and management of NR. Recently, the application of clinical models to predict outcomes has received increased attention in healthcare and medical research [4]. The models have the potential to significantly improve the accuracy of predicting cardiovascular risk following various interventions [5]. However, almost no predictive nomogram model has focused on coronary flow in AMI patients. In this study, we constructed an integrated and comprehensive nomogram model composed of demographics, medical history, and biochemical features and assessed the discrimination and calibration of a developed model of the NR phenomenon in AMI patients after pPCI in an attempt to identify and potentially prevent impaired reperfusion as quickly as possible.

## 2. Methods

### 2.1 Study Design

This was a single-center, retrospective, observational study. From January 2016 to December 2021, consecutive AMI patients admitted to the cardiology department of Xi’an Jiaotong University First Affiliated Hospital were enrolled. The inclusion criteria included a confirmed diagnosis of AMI, which was defined according to the electrocardiograms (ECGs), blood tests, and coronary angiography, according to the American College of Cardiology [6]. The exclusion criteria were (1) severe systemic disease, including but not limited to shock, cardiac arrest, malignant arrhythmias, coma, malignant tumor, respiratory failure requiring ventilatory support, renal failure requiring urgent dialysis, and bacterial sepsis with hemodynamic instability; (2) unwillingness to participate; (3) the patient was over the age of 75 years.

Medical records were collected from the Biobank Xi’an Jiaotong University First Affiliated Hospital. Written informed consent was obtained from all participants with ethical committee approval from the First Affiliated Hospital of Xi’an Jiaotong University, and the study was conducted in accordance with the Declaration of Helsinki.

### 2.2 Clinical Data Collection

Detailed medical histories were collected from the admitted patients. Demographic (age, sex), medical history (hypertension, diabetes mellitus), and biochemical markers (routine blood tests, basic metabolic panel, and coagulation function studies) were evaluated immediately after the patient’s admission to the hospital and prior to percutaneous coronary intervention (PCI). NR was defined as the absence of effective myocardial tissue perfusion after coronary artery recanalization (thrombolysis in myocardial infarction (TIMI) flow grade = 0) without obvious spasm, dissection, and residual stenosis [7]. Every patient suspected of coronary artery spasm received an intracoronary injection of nitroprusside/nitroglycerin to determine whether the spasm persisted and altered blood flow. According to the angiographic studies, two cardiologists defined NR after pPCI independently.

### 2.3 Development and Assessment of the Nomogram

Demographics, medical history, and biochemical markers were evaluated using univariable logistic regression. Variables with *p* < 0.2 after the univariable logistic analyses were included in the multivariable logistic analysis with three selection procedures (forward, backward, and stepwise) and nomogram construction. The score formula for total points was calculated from the nomogram system. The nomogram was used to formulate the best-fit regression model with the minimum Akaike’s information criterion. Receiver operator characteristic (ROC) curve analysis was used to evaluate the nomogram performance prediction. The calibration of the nomogram was assessed via calibration curves, and its goodness-of-fit was evaluated by the Hosmer–Lemeshow test. A decision curve analysis (DCA) was used to assess the clinical usefulness of the nomogram.

### 2.4 Statistical Analysis

R software (version 4.2.1, R Foundation for Statistical Computing, Vienna, Austria) with caret, rms, pROC, calibrate, rmda, and dca packages was used to perform all statistical analyses. Continuous variables were translated into categorical variables according to the standard normal range. All count data were expressed as rate (%). Univariate logistic regression and multivariate logistic regression were used to select risk factors.

A *p*-value < 0.05 was considered statistically significant.

## 3. Results

### 3.1 General Characteristics

**Fig. 1.**
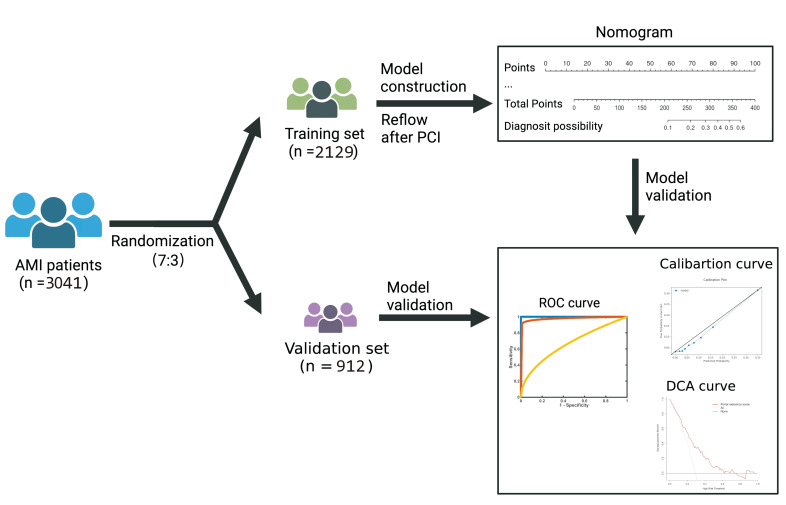
**Data analysis workflow**. AMI, acute myocardial infarction; PCI, percutaneous coronary intervention; ROC, receiver operator characteristic; DCA, decision curve analysis; AUC, area under the curve.

The study consisted of 3041 AMI patients who underwent PCI. These patients were divided into the training set (2129 (70.0%) and the validation set (912 (30.0%)) (Fig. 1). The clinical data, including medical history, examination, laboratory data, and information on cardiac angiographic procedures, are summarized in **Supplementary Table 1**. The clinical factors for predictors used in the nomogram are included in Table 1. The mean age was 62 in the training set and 62 in the validation set. Males accounted for 80.4% and 80.6% in the training and validation set, respectively. A total of 1626 (76.4%) were diagnosed with acute ST-segment elevated myocardial infarction (STEMI) in the training set and 699 (76.6%) in the validation set. In the two sets, 557 and 270 patients suffered from anterior, 370 and 168 patients from inferior posterior, 343 and 133 patients from high lateral, and 356 and 128 patients from inferior wall and right ventricle MI** (Supplementary Table 1)**. The NR event was 238 in the training set and 87 in the validation set. The level of N-terminal prohormone B-type natriuretic peptide (NT-proBNP), creatinine (Ccr), basophil count (BASO), hemoglobin (Hb), and D-dimer in NR patients was statistically significantly different in the reflow group both in the training set and validation set (Table 1).

**Table 1.** **Clinical characteristics of the patients selected as predictors for the nomogram**.

	Training set					Validation set			
	Level	All	Reflow	No reflow	*p* value	All	Reflow	No reflow	*p* value
	(0 = no, 1 = yes)	n = 2129	n = 1891	n = 238		n = 912	n = 825	n = 87	
Smoking (N (%))	0	904 (42.5)	789 (41.7)	115 (48.3)	0.061	382 (41.9)	342 (41.5)	40 (46.0)	0.485
	1	1225 (57.5)	1102 (58.3)	123 (51.7)		530 (58.1)	483 (58.5)	47 (54.0)	
NT-proBNP (median [IQR])		9.38 [7.63, 11.06]	9.29 [7.49, 10.88]	10.37 [8.54, 12.23]	<0.001	9.34 [7.43, 10.93]	9.22 [7.34, 10.86]	10.45 [8.76, 11.78]	<0.001
UA (N (%))	0	1830 (86.0)	1643 (86.9)	187 (78.6)	0.001	779 (85.4)	710 (86.1)	69 (79.3)	0.124
	1	299 (14.0)	248 (13.1)	51 (21.4)		133 (14.6)	115 (13.9)	18 (20.7)	
Ccr (N (%))	0	1937 (91.0)	1743 (92.2)	194 (81.5)	<0.001	832 (91.2)	758 (91.9)	74 (85.1)	0.050
	1	192 (9.0)	148 (7.8)	44 (18.5)		80 (8.8)	67 (8.1)	13 (14.9)	
TSH (N (%))	0	1994 (93.7)	1775 (93.9)	219 (92.0)	0.336	871 (95.5)	790 (95.8)	81 (93.1)	0.387
	1	135 (6.3)	116 (6.1)	19 (8.0)		41 (4.5)	35 (4.2)	6 (6.9)	
Hb (N (%))	0	1955 (91.8)	1764 (93.3)	191 (80.3)	<0.001	858 (94.1)	785 (95.2)	73 (83.9)	<0.001
	1	174 (8.2)	127 (6.7)	47 (19.7)		54 (5.9)	40 (4.8)	14 (16.1)	
D-dimer (N (%))	0	1658 (77.9)	1510 (79.9)	148 (62.2)	<0.001	696 (76.3)	639 (77.5)	57 (65.5)	0.018
	1	471 (22.1)	381 (20.1)	90 (37.8)		216 (23.7)	186 (22.5)	30 (34.5)	
PT (N (%))	0	34 (1.6)	33 (1.7)	1 (0.4)	0.207	17 (1.9)	16 (1.9)	1 (1.1)	0.919
	1	2095 (98.4)	1858 (98.3)	237 (99.6)		895 (98.1)	809 (98.1)	86 (98.9)	
TT (N (%))	0	1911 (89.8)	1692 (89.5)	219 (92.0)	0.269	801 (87.8)	720 (87.3)	81 (93.1)	0.159
	1	218 (10.2)	199 (10.5)	19 (8.0)		111 (12.2)	105 (12.7)	6 (6.9)	
BASO (N (%))	0	2110 (99.1)	1879 (99.4)	231 (97.1)	0.001	904 (99.1)	818 (99.2)	86 (98.9)	0.001
	1	19 (0.9)	12 (0.6)	7 (2.9)		8 (0.9)	7 (0.8)	1 (1.1)	
NEUBC (N (%))	0	862 (40.5)	738 (39.0)	124 (52.1)	<0.001	338 (37.1)	300 (36.4)	38 (43.7)	0.22
	1	1267 (59.5)	1153 (61.0)	114 (47.9)		574 (62.9)	525 (63.6)	49 (56.3)	
MONBC (N (%))	0	1704 (80.0)	1521 (80.4)	183 (76.9)	0.229	710 (77.9)	651 (78.9)	59 (67.8)	0.025
	1	425 (20.0)	370 (19.6)	55 (23.1)		202 (22.1)	174 (21.1)	28 (32.2)	
RDW.CV (N (%))	0	2037 (95.7)	1826 (96.6)	211 (88.7)	<0.001	867 (95.1)	787 (95.4)	80 (92.0)	0.251
	1	92 (4.3)	65 (3.4)	27 (11.3)		45 (4.9)	38 (4.6)	7 (8.0)	
MCHC (N (%))	0	2021 (94.9)	1789 (94.6)	232 (97.5)	0.081	850 (93.2)	764 (92.6)	86 (98.9)	0.048
	1	108 (5.1)	102 (5.4)	6 (2.5)		62 (6.8)	61 (7.4)	1 (1.1)	

NT-proBNP, N-terminal prohormone B-type natriuretic peptide; Ccr, creatinine; TSH, thyroid stimulating hormone; Hb, hemoglobin; PT, prothrombin time; TT, thrombin time; BASO, basophil count; NEUBC, neutrophil count; MONBC, monocyte count; RDW.CV, red blood cell distribution width; MCHC, mean corpuscular hemoglobin concentration; UA, uric acid; N, the value of percentage; n, numbers; IQR, interquartile range.

### 3.2 Nomogram Construction

**Fig. 2.**
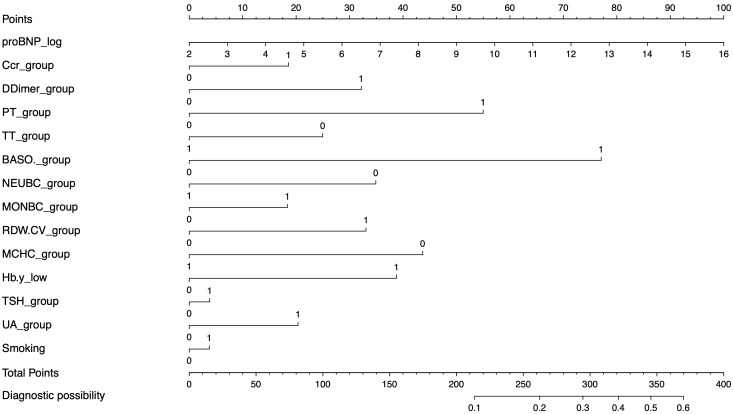
**The nomogram for the prediction of reflow in AMI patients after PCI**. proBNP, prohormone B-type natriuretic peptide; Ccr, creatinine; PT, prothrombin time; TT, thrombin time; BASO, basophil count; NEUBC, neutrophil count; MONBC, monocyte count; RDW.CV, red blood cell distribution width; MCHC, mean corpuscular hemoglobin concentration; Hb, hemoglobin; UA, uric acid; TSH, thyroid stimulating hormone; AMI, acute myocardial infarction; PCI, percutaneous coronary intervention. The value “1” stands for above the standard normal range, and “0” means in the standard normal range.

From the multivariate analyses with three selection procedures (stepwise, forward, and backward), we obtained the best-fit model, which contains 14 variables from the backward selection process (Fig. 2). Among these variables, NT-proBNP (OR 1.25, 95% CI 1.18 to 1.33, *p* = 0.003), Hb (OR 3.42, 95% CI 2.37 to 4.93, *p* = 0.015), D-dimer (OR 2.31, 95% CI 1.72 to 3.03, *p* < 0.001), BASO (OR 4.74, 95% CI 1.85 to 12.18, *p* = 0.003), neutrophil count (NEUBC) (OR 0.59, 95% CI 0.45 to 0.77, *p* < 0.001), and red blood cell distribution width (RDW.CV) (OR 3.59, 95% CI 2.25 to 5.75, *p* = 0.003) were independently associated with NR after pPCI (Table 2). This nomogram is displayed in Fig. 2. The nomogram formula, which could be used to calculate the total point, is as follows:

score = 7.14 × proBNP + 23.99 × Ccr + 33.84 × D-dimer + 63.89 × PT – 17.90 × TT + 82.54 × BASO –35.90 × NEUBC + 13.10 × MONBC + 44.78 × RDW.CV – 28.22 × MCHC + 29.92 × Hb – 0.60 × TSH + 14.77 × UA + 4.35 × smoking + 65.28

**Table 2.** **Selected variables as predictors for the nomogram according to the multivariable logistic analysis**.

Variables	Univariate analysis			Multivariate analysis		
	OR	95% CI	*p* value	OR	95% CI	*p* value
NT-proBNP	1.25	1.18–1.33	<0.001	1.14	1.58–1.82	<0.001
UA	1.81	1.29–2.53	<0.001	1.3	1.14–2.52	0.19
Ccr	2.67	1.85–3.86	<0.001	1.53	1.18–2.89	0.06
TSH	1.33	0.8–2.2	0.27	0.99	0.67–1.93	0.97
Hb	3.42	2.37–4.93	<0.001	1.7	0.01–0.02	0.02
D-dimer	2.31	1.72–3.03	<0.001	1.82	2.29–4.38	<0.001
PT	4.21	0.57–30.84	0.16	3.11	0.1–5.31	0.27
TT	0.74	0.45–1.21	0.23	0.73	2.57–7.29	0.23
BASO	4.74	1.85–12.18	<0.001	4.34	0.2–1.4	<0.001
NEUBC	0.59	0.45–0.77	<0.001	0.53	0.91–1.69	<0.001
MONBC	1.24	0.9–1.7	0.2	1.26	1.58–3.26	0.21
RDW.CV	3.59	2.25–5.75	<0.001	2.22	0.36–1.05	<0.001
MCHC	0.45	0.2–1.05	0.06	0.61	0.72–4.01	0.25
Smoking	0.77	0.58–1	0.05	0.93	0.85–1.53	0.60

NT-proBNP, N-terminal prohormone B-type natriuretic peptide; Ccr, creatinine; TSH, thyroid stimulating hormone; Hb, hemoglobin; PT, prothrombin time; TT, thrombin time; BASO, basophil count; NEUBC, neutrophil count; MONBC, monocyte count; RDW.CV, red blood cell distribution width; MCHC, mean corpuscular hemoglobin concentration; UA, uric acid; OR, odds ratio; CI, confidence interval.

### 3.3 Evaluation of the Nomogram

**Fig. 3.**
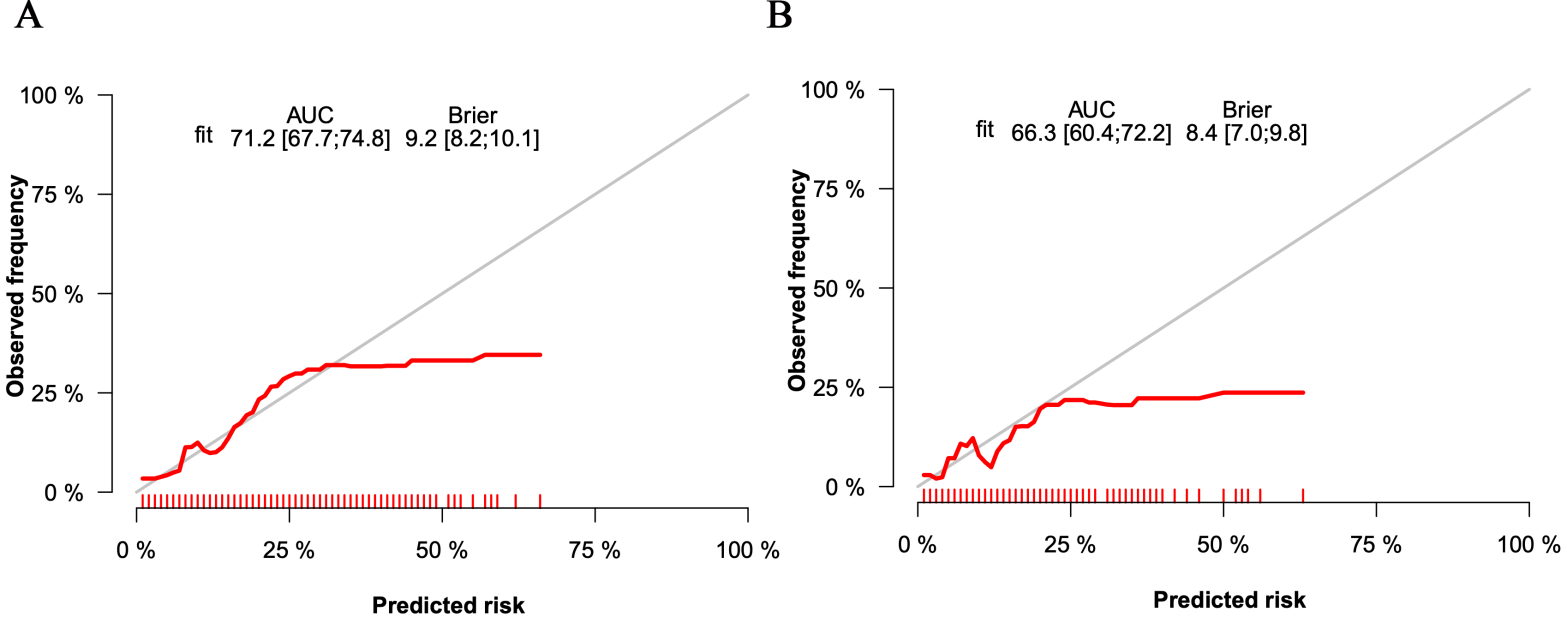
**The calibration curves of the nomogram for the training set (A) and the validation set (B)**. AUC, area under the curve.

**Fig. 4.**
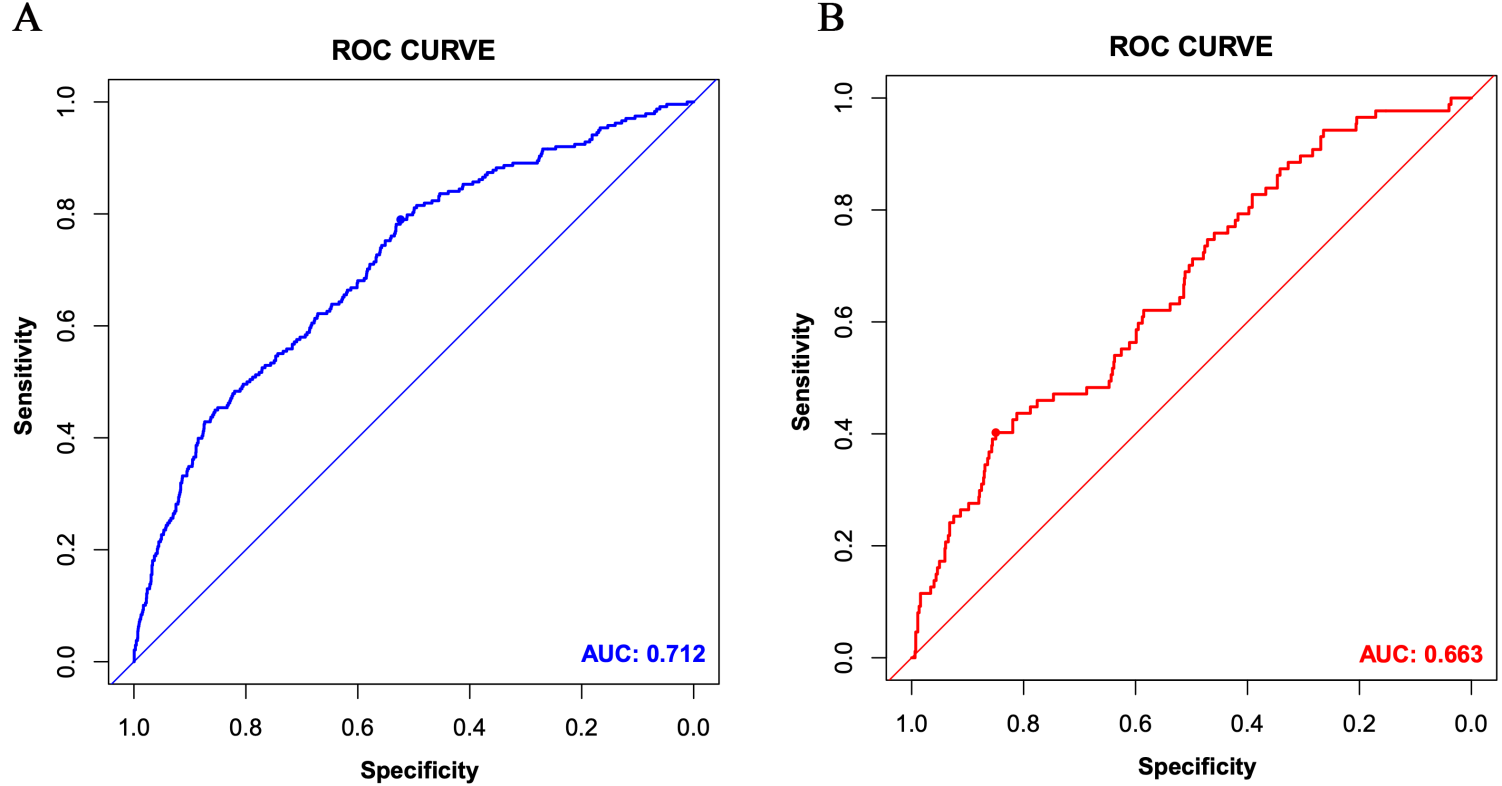
**The receiver operating characteristic (ROC) curves of the nomogram for the training set (A) and the validation set (B)**. AUC, area under the curve.

**Fig. 5.**
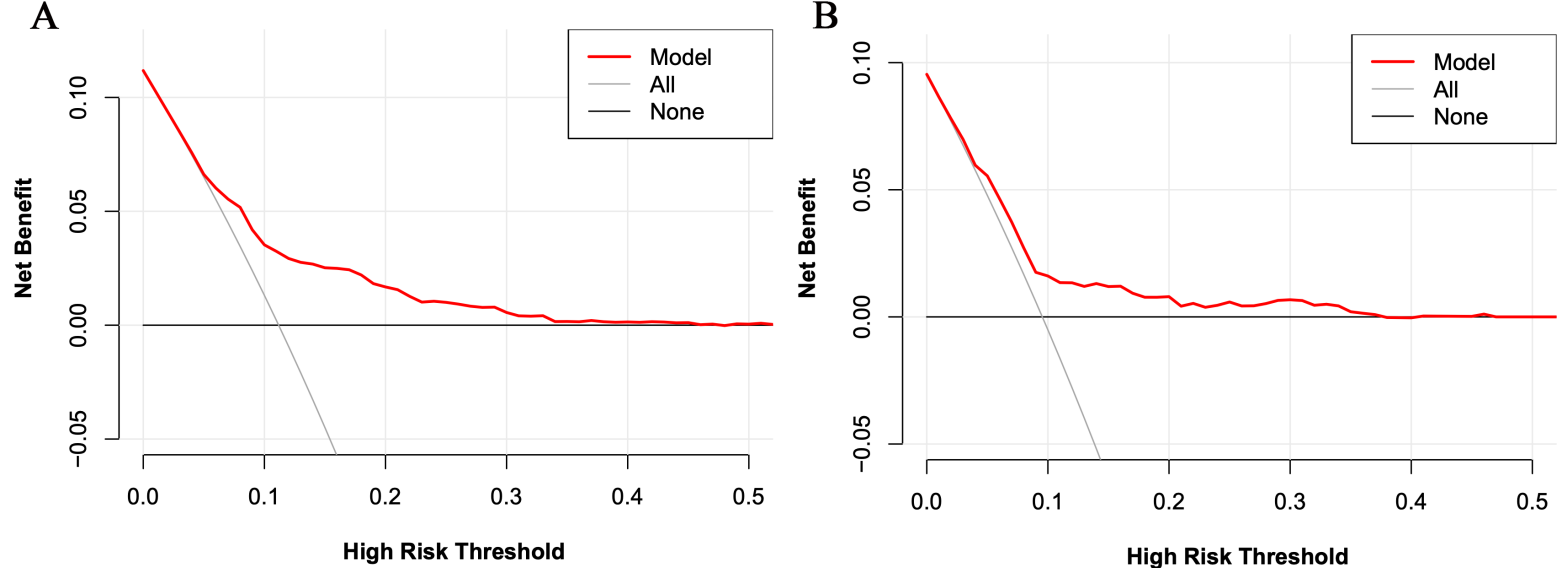
**The decision curve analysis for the risk model for the training set (A) and the validation set (B)**.

The C-index in the training set was 0.712, indicating that the prediction model was valuable in clinical practice (Fig. 3A). The *p*-value of the Hosmer–Lemeshow test was 0.211 (>0.05), reflecting a good prediction accuracy. Fig. 4A displays the ROC curve (area under the curve, AUC = 0.712, 95% CI 0.677 to 0.748). The DCA curve for the training set is shown in Fig. 5A, suggesting that the nomogram could provide an overall net benefit for predicting NR after pPCI.

In the validation set, the C-index was 0.663. Fig. 3B shows the calibration curve. Fig. 4B shows the ROC curve of the validation set (AUC 0.663, 95% CI 0.604 to 0.722). The DCA curve is displayed in Fig. 5B. These results suggest that the nomogram had acceptable discrimination and prediction accuracy in the validation set.

## 4. Discussion

This study developed a prediction model for the NR phenomenon in AMI patients after pPCI. Using this clinical nomogram, eight significant predictors were screened. We found that impaired cardiac and renal function, increased uric acid (UA) and thyroid stimulating hormone (TSH) levels, a hypercoagulable state, and abnormal blood cell counts were predictors of no-reflow.

The pathophysiology of the NR phenomenon is not fully understood, and various mechanisms have been suggested to explain this phenomenon. In an experimental study, neutrophil accumulation, coagulation cascade, and reactive oxygen species-induced endothelial dysfunction were observed to increase microvascular constriction [8]. In addition, diabetes, hypercholesterolemia, metabolic dysfunction, and increased reperfusion injury were noted in animal models by augmenting endothelial oxidative stress [9]. Furthermore, certain medications, such as sodium-dependent glucose transporters 2 (SGLT2) inhibitors, were observed to modulate microcirculation through anti-inflammatory effects, potentially enhancing outcomes for AMI patients [10]. Noteworthy studies in type 2 diabetes mellitus (T2DM) individuals with AMI demonstrated that SGLT2 inhibitors reduced the risk of adverse cardiovascular events during both index hospitalization and long-term follow-up [11]. Interestingly, these inhibitors exhibited a capacity to mitigate in-stent restenosis-related events post-AMI [12], possibly through pleiotropic effects on coronary fibrous cap thickness, consequently reducing major adverse cardiac events (MACEs) in higher-risk patients [13].

Apart from the above “classic” metabolic risk factors, a few novel factors may also play significant roles in NR. Increased UA levels, which represent the end product of purine metabolism, are associated with increased mortality in AMI patients [14]. Yildiz *et al*. [15] found that elevated UA levels were an independent predictor for insufficient coronary blood flow in patients during normal coronary angiography (0% stenosis), indicating that impaired coronary microvascular regulation may cause NR. Our nomogram further demonstrated that increased UA may account for NR, most likely due to an increased inflammatory response [16]. Elevated TSH levels, which are associated with hypothyroidism and decreased thyroid hormone levels, were also shown to be predictive of NR in this model. This suggests that decreased thyroid metabolism and catecholamine levels manifested by elevated TSH feedback may affect coronary blood flow in AMI patients.

The coagulation system is vital in the occurrence and progression of thrombosis in AMI [17]. Both increased prothrombin time (PT) and D-dimer levels were observed in the NR group, while thrombin time was decreased, all of which contribute to a hypercoagulable state in NR. Several studies showed increased serum D-dimer levels, which reflects the activation of the coagulation system resulting in thrombosis [18, 19] and serves as an indirect prediction of the thrombotic mass size available for fibrinolysis [20], indirectly reflecting the size of thrombus formation [21]. D-dimer levels are significantly higher in patients treated within 12 h of symptom onset and with higher TIMI thrombus scores [22]. The thrombus burden leading to vascular emboli plays an important role in the pathophysiology of NR after primary PCI and occurs in half of the MI patients.

Several easily calculated hematological indices, including the NEUBC, red blood cell distribution width, mean platelet volume (MPV), neutrophil–lymphocyte ratio (NLR), platelet–lymphocyte ratio (PLR), and RDW–platelet ratio (RPR), is of prognostic value in STEMI [23] and may be associated with the pathogenesis of NR. In our nomogram, NR was associated with increased BASO, RDWCV, and MONBC and decreased NEUBC, MCHC, and Hb. Increased RDW.CV represents reduced erythrocyte deformability, which may cause microvascular blood flow resistance, and has been shown to be an independent predictor of coronary thrombus burden [24, 25]. Similar to our results, Chang *et al*. [26] found that RDW.CV was also an independent predictor for long-term MACEs in STEMI patients after pPCI. The association of increased hypersensitive C-reactive protein (hs-CRP) and RDW.CV levels in NR [27] suggest that inflammatory and oxidative stress could be one of the mechanical factors that links elevated RDW.CV and NR by damaging the vessel wall [28]. In addition to decreased hemoglobin concentrations seen in our model, RDW.CV levels seen in anemia predict an even worse outcome in patients with acute coronary syndromes [29] and are a potential risk factor for NR [30]. Additionally, increased circulating monocytes could induce the production of chemotactic factors, such as monocyte-chemoattractant-protein-1 (MCP-1) and interleukin-8 (IL-8), which induce the expression of tissue factors, superoxide anions, and exerts prothrombotic effects [31]. Furthermore, mechanical obstruction of the microvasculature after monocyte-induced neutrophil accumulation might also contribute to the occurrence of NR [32].

In contemporary healthcare and medical research, there has been a discernible surge in interest in applying clinical predictive models [4]. Supervised learning has emerged as a particularly apt approach for tasks characterized by well-defined objectives, given its amenability to facile quantification through diverse metrics, thereby facilitating the straightforward evaluation of accuracy and efficacy [33, 34]. Reinforcement learning (RL) [35], characterized by its adaptive nature, can accommodate dynamic and evolving environments, rendering it well-suited for scenarios where optimal strategies may undergo temporal evolution. Furthermore, RL exhibits the advantage of being trainable in simulated environments, thereby mitigating the reliance on extensive real-world datasets [36]. The prospective utilization of these machine learning models in future research holds potential for discerning predictors, constructing expansive and diversified patient models, and enhancing accuracy in cardiovascular risk prediction [5].

The nomogram can become a simple and intuitive mathematical model [37]. After calculating the predicted risk and relative scores, patients with a point score of 322 would have a more than 50% possibility of developing severe NR. A higher score indicates the need for intensive care, hemodynamic monitoring, and immediate evaluation of patients to prevent potentially impaired reperfusion.

Our study has several limitations that need to be acknowledged. First, being a single-center study, the cohort samples might only represent the population of west China. To enhance the generalizability of our findings, we plan to conduct an additional validation assessment in a multi-center study. Second, we observed relatively low C-index and AUC values in the validation set. We intend to enlarge the sample size and explore alternative modeling techniques to address this in future studies. Since our focus in this study was primarily on biomedical parameters, we did not assess the correlation between NR and other factors such as criminal vessels, balloon-to-door time, or the use of glycoprotein IIb/IIIa inhibitors. Moreover, we recognize the need for detailed information on PCI and angiographic procedures in future research to gain a comprehensive understanding. Lastly, TIMI flow grade was the primary “standard” assessment for NR in this study. In future studies, we plan to expand our evaluation by incorporating other criteria, including corrected thrombolysis in myocardial infarction frame count (CTFC) and myocardial blush grade (MBG) assessment, and explore different deep learning models, to determine better predictors and provide a more comprehensive analysis of NR.

## 5. Conclusions

In conclusion, a nomogram to predict the no-reflow phenomenon in AMI patients after pPCI was developed and validated in west China. We hope this nomogram can be used for NR risk assessment and clinical decision-making in AMI patients, which may more rapidly prevent potentially impaired reperfusion associated with NR following PCI during an AMI.

## Data Availability

The datasets used or analyzed during the current study are available from the corresponding authors on reasonable request.

## Supplementary Material

Supplementary material associated with this article can be found, in the online version, at https://doi.org/10.31083/j.rcm2505151.
