# Supplementary material for: A Prediction Nomogram for No-Reflow in Acute Myocardial Infarction Patients after Primary Percutaneous Coronary Intervention
Source: Rev Cardiovasc Med. 2024 Apr 30;25(5):151. doi: 10.31083/j.rcm2505151 (PMC11267190; doi:10.31083/j.rcm2505151)
Supplement: Supplementary file 1 [file 2153-8174-25-5-151-s1.docx]

**Supplemental Table 1. Medical history, examination, laboratory data and information on cardiac angiographic procedures in both training and validation sets.**

|  |  | Training Set | | | | Validation Set | | | |
| --- | --- | --- | --- | --- | --- | --- | --- | --- | --- |
| Variables | **Level*** | **All** | **Reflowed** | **No-reflow** | **P value** | **All** | **Reflowed** | **No-reflow** | **P value** |
|  |  | (n=2129) | (n=1891) | (n=238) |  | (n=912) | (n=825) | (n=87) |  |
| Smoking (%) | No | 904 (42.5) | 789 (41.7) | 115 (48.3) | 0.061 | 382 (41.9) | 342 (41.5) | 40 (46.0) | 0.485 |
|  | Yes | 1225 (57.5) | 1102 (58.3) | 123 (51.7) |  | 530 (58.1) | 483 (58.5) | 47 (54.0) |  |
| Age (median [IQR]) |  | 62.00 [54.00, 71.00] | 62.00 [54.00, 70.00] | 66.00 [56.00, 77.00] | <0.001 | 62.00 [53.00, 70.00] | 62.00 [53.00, 70.00] | 65.00 [55.50, 75.00] | 0.024 |
| Sex (%) | Male | 1712 (80.4) | 1539 (81.4) | 173 (72.7) | 0.002 | 735 (80.6) | 664 (80.5) | 71 (81.6) | 0.913 |
|  | Female | 417 (19.6) | 352 (18.6) | 65 (27.3) |  | 177 (19.4) | 161 (19.5) | 16 (18.4) |  |
| STEMI(%) |  | 1626(76.4) | 1445(76.4) | 181(76.1) | 0.935 | 699(76.6) | 632(76.6) | 67(76.8) | 0.999 |
|  | Anterior | 557 | 495 | 62 |  | 270 | 244 | 26 |  |
|  | Inferior posterior | 370 | 329 | 41 |  | 168 | 156 | 16 |  |
|  | High lateral | 343 | 305 | 38 |  | 133 | 120 | 13 |  |
|  | Inferior and right ventricle | 356 | 316 | 40 |  | 128 | 112 | 12 |  |
| NSTEMI(%) |  | 503 (23.6) | 446(23.5) | 57(23.9) |  | 213(23.4) | 193(23.4) | 20 (23.2) |  |
| DM (%) | No | 1544 (72.5) | 1384 (73.2) | 160 (67.2) | 0.062 | 671 (73.6) | 605 (73.3) | 66 (75.9) | 0.703 |
|  | Yes | 585 (27.5) | 507 (26.8) | 78 (32.8) |  | 241 (26.4) | 220 (26.7) | 21 (24.1) |  |
| Hypertension (%) | No | 1025 (48.1) | 925 (48.9) | 100 (42.0) | 0.053 | 428 (46.9) | 388 (47.0) | 40 (46.0) | 0.941 |
|  | Yes | 1104 (51.9) | 966 (51.1) | 138 (58.0) |  | 484 (53.1) | 437 (53.0) | 47 (54.0) |  |
| Systolic pressure (median [IQR]) |  | 121.00 [107.00, 137.00] | 121.00 [107.00, 137.00] | 122.50 [108.25, 138.00] | 0.339 | 121.00 [106.00, 138.00] | 121.00 [106.00, 138.00] | 119.00 [105.50, 139.50] | 0.714 |
| Diastolic pressure (median [IQR]) |  | 76.00 [67.00, 87.00] | 76.00 [68.00, 87.00] | 75.00 [65.00, 86.00] | 0.162 | 76.00 [66.00, 87.00] | 76.00 [67.00, 88.00] | 74.00 [63.00, 83.50] | 0.052 |
| Respiratory frequency (median [IQR]) |  | 19.00 [18.00, 20.00] | 19.00 [18.00, 20.00] | 19.00 [18.00, 20.00] | 0.584 | 19.00 [18.00, 20.00] | 19.00 [18.00, 20.00] | 19.00 [18.00, 20.00] | 0.29 |
| Body temperature (median [IQR]) |  | 36.30 [36.00, 36.50] | 36.30 [36.00, 36.50] | 36.30 [36.10, 36.60] | 0.232 | 36.30 [36.10, 36.60] | 36.30 [36.00, 36.60] | 36.30 [36.20, 36.50] | 0.209 |
| Hs_cTnT (%) | No | 149 (7.0) | 133 (7.0) | 16 (6.7) | 0.966 | 81 (8.9) | 77 (9.3) | 4 (4.6) | 0.201 |
|  | Yes | 1980 (93.0) | 1758 (93.0) | 222 (93.3) |  | 831 (91.1) | 748 (90.7) | 83 (95.4) |  |
| HbA1c (%) | No | 1395 (65.5) | 1249 (66.0) | 146 (61.3) | 0.172 | 606 (66.4) | 546 (66.2) | 60 (69.0) | 0.686 |
|  | Yes | 734 (34.5) | 642 (34.0) | 92 (38.7) |  | 306 (33.6) | 279 (33.8) | 27 (31.0) |  |
| proBNP(median [IQR]) |  | 9.38 [7.63, 11.06] | 9.29 [7.49, 10.88] | 10.37 [8.54, 12.23] | <0.001 | 9.34 [7.43, 10.93] | 9.22 [7.34, 10.86] | 10.45 [8.76, 11.78] | <0.001 |
| TSH (%) | No | 1994 (93.7) | 1775 (93.9) | 219 (92.0) | 0.336 | 871 (95.5) | 790 (95.8) | 81 (93.1) | 0.387 |
|  | Yes | 135 (6.3) | 116 (6.1) | 19 (8.0) |  | 41 (4.5) | 35 (4.2) | 6 (6.9) |  |
| T3(%) | No | 2125 (99.8) | 1887 (99.8) | 238 (100.0) | 1 | 908 (99.6) | 823 (99.8) | 85 (97.7) | 0.056 |
|  | Yes | 4 (0.2) | 4 (0.2) | 0 (0.0) |  | 4 (0.4) | 2 (0.2) | 2 (2.3) |  |
| T4 (%) | No | 2123 (99.7) | 1885 (99.7) | 238 (100.0) | 0.825 | 906 (99.3) | 820 (99.4) | 86 (98.9) | 1 |
|  | Yes | 6 (0.3) | 6 (0.3) | 0 (0.0) |  | 6 (0.7) | 5 (0.6) | 1 (1.1) |  |
| TC (%) | No | 1973 (92.7) | 1746 (92.3) | 227 (95.4) | 0.117 | 852 (93.4) | 766 (92.8) | 86 (98.9) | 0.055 |
|  | Yes | 156 (7.3) | 145 (7.7) | 11 (4.6) |  | 60 (6.6) | 59 (7.2) | 1 (1.1) |  |
| TG (%) | No | 1287 (60.5) | 1137 (60.1) | 150 (63.0) | 0.429 | 544 (59.6) | 481 (58.3) | 63 (72.4) | 0.015 |
|  | Yes | 842 (39.5) | 754 (39.9) | 88 (37.0) |  | 368 (40.4) | 344 (41.7) | 24 (27.6) |  |
| LDL (%) | No | 1747 (82.1) | 1547 (81.8) | 200 (84.0) | 0.451 | 754 (82.7) | 676 (81.9) | 78 (89.7) | 0.097 |
|  | Yes | 382 (17.9) | 344 (18.2) | 38 (16.0) |  | 158 (17.3) | 149 (18.1) | 9 (10.3) |  |
| HDL (%) | No | 2056 (96.6) | 1828 (96.7) | 228 (95.8) | 0.613 | 889 (97.5) | 803 (97.3) | 86 (98.9) | 0.618 |
|  | Yes | 73 (3.4) | 63 (3.3) | 10 (4.2) |  | 23 (2.5) | 22 (2.7) | 1 (1.1) |  |
| Cys (%) | No | 1518 (71.3) | 1368 (72.3) | 150 (63.0) | 0.004 | 634 (69.5) | 585 (70.9) | 49 (56.3) | 0.007 |
|  | Yes | 611 (28.7) | 523 (27.7) | 88 (37.0) |  | 278 (30.5) | 240 (29.1) | 38 (43.7) |  |
| Homocys (%) | No | 1170 (55.0) | 1047 (55.4) | 123 (51.7) | 0.313 | 501 (54.9) | 462 (56.0) | 39 (44.8) | 0.06 |
|  | Yes | 959 (45.0) | 844 (44.6) | 115 (48.3) |  | 411 (45.1) | 363 (44.0) | 48 (55.2) |  |
| Plt (%) | No | 2069 (97.2) | 1842 (97.4) | 227 (95.4) | 0.115 | 882 (96.7) | 797 (96.6) | 85 (97.7) | 0.819 |
|  | Yes | 60 (2.8) | 49 (2.6) | 11 (4.6) |  | 30 (3.3) | 28 (3.4) | 2 (2.3) |  |
| ApoA (%) | No | 2112 (99.2) | 1875 (99.2) | 237 (99.6) | 0.757 | 910 (99.8) | 823 (99.8) | 87 (100.0) | 1 |
|  | Yes | 17 (0.8) | 16 (0.8) | 1 (0.4) |  | 2 (0.2) | 2 (0.2) | 0 (0.0) |  |
| ApoB(%) | No | 1862 (87.5) | 1647 (87.1) | 215 (90.3) | 0.187 | 798 (87.5) | 716 (86.8) | 82 (94.3) | 0.067 |
|  | Yes | 267 (12.5) | 244 (12.9) | 23 (9.7) |  | 114 (12.5) | 109 (13.2) | 5 (5.7) |  |
| ApoE (%) | No | 1712 (80.4) | 1520 (80.4) | 192 (80.7) | 0.984 | 763 (83.7) | 686 (83.2) | 77 (88.5) | 0.257 |
|  | Yes | 417 (19.6) | 371 (19.6) | 46 (19.3) |  | 149 (16.3) | 139 (16.8) | 10 (11.5) |  |
| Lpa(%) | No | 1535 (72.1) | 1366 (72.2) | 169 (71.0) | 0.748 | 671 (73.6) | 612 (74.2) | 59 (67.8) | 0.249 |
|  | Yes | 594 (27.9) | 525 (27.8) | 69 (29.0) |  | 241 (26.4) | 213 (25.8) | 28 (32.2) |  |
| Ccr (%) | No | 1937 (91.0) | 1743 (92.2) | 194 (81.5) | <0.001 | 832 (91.2) | 758 (91.9) | 74 (85.1) | 0.052 |
|  | Yes | 192 (9.0) | 148 (7.8) | 44 (18.5) |  | 80 (8.8) | 67 (8.1) | 13 (14.9) |  |
| UA (%) | No | 1830 (86.0) | 1643 (86.9) | 187 (78.6) | 0.001 | 779 (85.4) | 710 (86.1) | 69 (79.3) | 0.124 |
|  | Yes | 299 (14.0) | 248 (13.1) | 51 (21.4) |  | 133 (14.6) | 115 (13.9) | 18 (20.7) |  |
| DDimer (%) | No | 1658 (77.9) | 1510 (79.9) | 148 (62.2) | <0.001 | 696 (76.3) | 639 (77.5) | 57 (65.5) | 0.018 |
|  | Yes | 471 (22.1) | 381 (20.1) | 90 (37.8) |  | 216 (23.7) | 186 (22.5) | 30 (34.5) |  |
| PTR (%) | No | 2029 (95.3) | 1812 (95.8) | 217 (91.2) | 0.002 | 857 (94.0) | 773 (93.7) | 84 (96.6) | 0.408 |
|  | Yes | 100 (4.7) | 79 (4.2) | 21 (8.8) |  | 55 (6.0) | 52 (6.3) | 3 (3.4) |  |
| PTA (%) | No | 2116 (99.4) | 1878 (99.3) | 238 (100.0) | 0.4 | 906 (99.3) | 819 (99.3) | 87 (100.0) | 0.92 |
|  | Yes | 13 (0.6) | 13 (0.7) | 0 (0.0) |  | 6 (0.7) | 6 (0.7) | 0 (0.0) |  |
| APTT (%) | No | 204 (9.6) | 178 (9.4) | 26 (10.9) | 0.529 | 85 (9.3) | 79 (9.6) | 6 (6.9) | 0.533 |
|  | Yes | 1925 (90.4) | 1713 (90.6) | 212 (89.1) |  | 827 (90.7) | 746 (90.4) | 81 (93.1) |  |
| PT (%) | No | 34 (1.6) | 33 (1.7) | 1 (0.4) | 0.207 | 17 (1.9) | 16 (1.9) | 1 (1.1) | 0.919 |
|  | Yes | 2095 (98.4) | 1858 (98.3) | 237 (99.6) |  | 895 (98.1) | 809 (98.1) | 86 (98.9) |  |
| INR (%) | No | 2025 (95.1) | 1808 (95.6) | 217 (91.2) | 0.005 | 855 (93.8) | 772 (93.6) | 83 (95.4) | 0.662 |
|  | Yes | 104 (4.9) | 83 (4.4) | 21 (8.8) |  | 57 (6.2) | 53 (6.4) | 4 (4.6) |  |
| TT (%) | No | 1911 (89.8) | 1692 (89.5) | 219 (92.0) | 0.269 | 801 (87.8) | 720 (87.3) | 81 (93.1) | 0.159 |
|  | Yes | 218 (10.2) | 199 (10.5) | 19 (8.0) |  | 111 (12.2) | 105 (12.7) | 6 (6.9) |  |
| Fg (%) | No | 1530 (71.9) | 1383 (73.1) | 147 (61.8) | <0.001 | 658 (72.1) | 606 (73.5) | 52 (59.8) | 0.01 |
|  | Yes | 599 (28.1) | 508 (26.9) | 91 (38.2) |  | 254 (27.9) | 219 (26.5) | 35 (40.2) |  |
| FDP (%) | No | 1915 (89.9) | 1722 (91.1) | 193 (81.1) | <0.001 | 811 (88.9) | 734 (89.0) | 77 (88.5) | 1 |
|  | Yes | 214 (10.1) | 169 (8.9) | 45 (18.9) |  | 101 (11.1) | 91 (11.0) | 10 (11.5) |  |
| hs.CRP (%) | No | 908 (42.6) | 821 (43.4) | 87 (36.6) | 0.051 | 391 (42.9) | 361 (43.8) | 30 (34.5) | 0.121 |
|  | Yes | 1221 (57.4) | 1070 (56.6) | 151 (63.4) |  | 521 (57.1) | 464 (56.2) | 57 (65.5) |  |
| BASO (%) | No | 2110 (99.1) | 1879 (99.4) | 231 (97.1) | 0.001 | 904 (99.1) | 818 (99.2) | 86 (98.9) | 1 |
|  | Yes | 19 (0.9) | 12 (0.6) | 7 (2.9) |  | 8 (0.9) | 7 (0.8) | 1 (1.1) |  |
| EOS (%) | No | 2121 (99.6) | 1885 (99.7) | 236 (99.2) | 0.496 | 907 (99.5) | 820 (99.4) | 87 (100.0) | 1 |
|  | Yes | 8 (0.4) | 6 (0.3) | 2 (0.8) |  | 5 (0.5) | 5 (0.6) | 0 (0.0) |  |
| GR (%) | No | 865 (40.6) | 751 (39.7) | 114 (47.9) | 0.019 | 338 (37.1) | 302 (36.6) | 36 (41.4) | 0.447 |
|  | Yes | 1264 (59.4) | 1140 (60.3) | 124 (52.1) |  | 574 (62.9) | 523 (63.4) | 51 (58.6) |  |
| MON (%) | No | 2096 (98.4) | 1863 (98.5) | 233 (97.9) | 0.652 | 902 (98.9) | 816 (98.9) | 86 (98.9) | 1 |
|  | Yes | 33 (1.6) | 28 (1.5) | 5 (2.1) |  | 10 (1.1) | 9 (1.1) | 1 (1.1) |  |
| LY (%) | No | 2125 (99.8) | 1887 (99.8) | 238 (100.0) | 1 | 910 (99.8) | 823 (99.8) | 87 (100.0) | 1 |
|  | Yes | 4 (0.2) | 4 (0.2) | 0 (0.0) |  | 2 (0.2) | 2 (0.2) | 0 (0.0) |  |
| BASOC (%) | No | 2037 (95.7) | 1812 (95.8) | 225 (94.5) | 0.454 | 860 (94.3) | 779 (94.4) | 81 (93.1) | 0.793 |
|  | Yes | 92 (4.3) | 79 (4.2) | 13 (5.5) |  | 52 (5.7) | 46 (5.6) | 6 (6.9) |  |
| EOSC (%) | No | 2115 (99.3) | 1880 (99.4) | 235 (98.7) | 0.426 | 907 (99.5) | 820 (99.4) | 87 (100.0) | 1 |
|  | Yes | 14 (0.7) | 11 (0.6) | 3 (1.3) |  | 5 (0.5) | 5 (0.6) | 0 (0.0) |  |
| NEUBC (%) | No | 862 (40.5) | 738 (39.0) | 124 (52.1) | <0.001 | 338 (37.1) | 300 (36.4) | 38 (43.7) | 0.22 |
|  | Yes | 1267 (59.5) | 1153 (61.0) | 114 (47.9) |  | 574 (62.9) | 525 (63.6) | 49 (56.3) |  |
| MONBC (%) | No | 1704 (80.0) | 1521 (80.4) | 183 (76.9) | 0.229 | 710 (77.9) | 651 (78.9) | 59 (67.8) | 0.025 |
|  | Yes | 425 (20.0) | 370 (19.6) | 55 (23.1) |  | 202 (22.1) | 174 (21.1) | 28 (32.2) |  |
| LBC (%) | No | 2071 (97.3) | 1837 (97.1) | 234 (98.3) | 0.402 | 885 (97.0) | 798 (96.7) | 87 (100.0) | 0.167 |
|  | Yes | 58 (2.7) | 54 (2.9) | 4 (1.7) |  | 27 (3.0) | 27 (3.3) | 0 (0.0) |  |
| WBC (%) | No | 1135 (53.3) | 991 (52.4) | 144 (60.5) | 0.022 | 465 (51.0) | 417 (50.5) | 48 (55.2) | 0.479 |
|  | Yes | 994 (46.7) | 900 (47.6) | 94 (39.5) |  | 447 (49.0) | 408 (49.5) | 39 (44.8) |  |
| PCT (%) | No | 2086 (98.0) | 1855 (98.1) | 231 (97.1) | 0.408 | 891 (97.7) | 807 (97.8) | 84 (96.6) | 0.709 |
|  | Yes | 43 (2.0) | 36 (1.9) | 7 (2.9) |  | 21 (2.3) | 18 (2.2) | 3 (3.4) |  |
| P.LCR(%) | No | 1887 (88.6) | 1676 (88.6) | 211 (88.7) | 1 | 806 (88.4) | 731 (88.6) | 75 (86.2) | 0.625 |
|  | Yes | 242 (11.4) | 215 (11.4) | 27 (11.3) |  | 106 (11.6) | 94 (11.4) | 12 (13.8) |  |
| MPV(%) | No | 1864 (87.6) | 1654 (87.5) | 210 (88.2) | 0.815 | 798 (87.5) | 722 (87.5) | 76 (87.4) | 1 |
|  | Yes | 265 (12.4) | 237 (12.5) | 28 (11.8) |  | 114 (12.5) | 103 (12.5) | 11 (12.6) |  |
| PLT (%) | No | 2068 (97.1) | 1841 (97.4) | 227 (95.4) | 0.129 | 882 (96.7) | 797 (96.6) | 85 (97.7) | 0.819 |
|  | Yes | 61 (2.9) | 50 (2.6) | 11 (4.6) |  | 30 (3.3) | 28 (3.4) | 2 (2.3) |  |
| RDW.SD (%) | No | 2051 (96.3) | 1831 (96.8) | 220 (92.4) | 0.001 | 874 (95.8) | 792 (96.0) | 82 (94.3) | 0.622 |
|  | Yes | 78 (3.7) | 60 (3.2) | 18 (7.6) |  | 38 (4.2) | 33 (4.0) | 5 (5.7) |  |
| RDW.CV(%) | No | 2037 (95.7) | 1826 (96.6) | 211 (88.7) | <0.001 | 867 (95.1) | 787 (95.4) | 80 (92.0) | 0.251 |
|  | Yes | 92 (4.3) | 65 (3.4) | 27 (11.3) |  | 45 (4.9) | 38 (4.6) | 7 (8.0) |  |
| MCHC (%) | No | 2021 (94.9) | 1789 (94.6) | 232 (97.5) | 0.081 | 850 (93.2) | 764 (92.6) | 86 (98.9) | 0.048 |
|  | Yes | 108 (5.1) | 102 (5.4) | 6 (2.5) |  | 62 (6.8) | 61 (7.4) | 1 (1.1) |  |
| MCH(%) | No | 2026 (95.2) | 1797 (95.0) | 229 (96.2) | 0.518 | 863 (94.6) | 781 (94.7) | 82 (94.3) | 1 |
|  | Yes | 103 (4.8) | 94 (5.0) | 9 (3.8) |  | 49 (5.4) | 44 (5.3) | 5 (5.7) |  |
| MCV (%) | No | 1987 (93.3) | 1766 (93.4) | 221 (92.9) | 0.863 | 848 (93.0) | 768 (93.1) | 80 (92.0) | 0.862 |
|  | Yes | 142 (6.7) | 125 (6.6) | 17 (7.1) |  | 64 (7.0) | 57 (6.9) | 7 (8.0) |  |
| LPCVV (%) | No | 1141 (53.6) | 996 (52.7) | 145 (60.9) | 0.019 | 481 (52.7) | 423 (51.3) | 58 (66.7) | 0.009 |
|  | Yes | 988 (46.4) | 895 (47.3) | 93 (39.1) |  | 431 (47.3) | 402 (48.7) | 29 (33.3) |  |
| RBC (%) | No | 2051 (96.3) | 1831 (96.8) | 220 (92.4) | 0.001 | 874 (95.8) | 792 (96.0) | 82 (94.3) | 0.622 |
|  | Yes | 78 (3.7) | 60 (3.2) | 18 (7.6) |  | 38 (4.2) | 33 (4.0) | 5 (5.7) |  |
| Hb (%) | No | 196 (9.2) | 149 (7.9) | 47 (19.7) | <0.001 | 65 (7.1) | 49 (5.9) | 16 (18.4) | <0.001 |
|  | Yes | 1236 (58.1) | 1099 (58.1) | 137 (57.6) |  | 555 (60.9) | 502 (60.8) | 53 (60.9) |  |

*In biochemical parameters, no represents within the normal range, and yes represents outside the normal range.
